# Impact of Non-Alcoholic Fatty Liver Disease on Sepsis Inpatient Outcomes: A Nationwide Sample Analysis (2000–2019)

**DOI:** 10.3390/jcm13195737

**Published:** 2024-09-26

**Authors:** Xiuhong Lyu, Bolun Liu, Yiting Li, Yichen Wang, John Miskovsky, Melissa Gaitanis, Kittichai Promrat, Wen-Chih Wu

**Affiliations:** 1 Department of Epidemiology, School of Public Health, Brown University, 121 South Main Street, Providence, RI 02903, USA; xiuhonglyu@outlook.com; 2 Department of Adult Medicine, Brockton Neighborhood Health Center, 63 Main Street, Brockton, MA 02301, USA; 3 Department of Hospital Internal Medicine, Mayo Clinic Health System, 1025 Marsh Street, Mankato, MN 56001, USA; liu.bolun@mayo.edu; 4 Division of Gastroenterology and Hepatology, University of New–Mexico Health Science Center, 2500 Marble Ave., Albuquerque, NM 87106, USA; 5 Division of Hospital Medicine, The Hospital of the University of Pennsylvania, Penn Presbyterian Medical Center, 3400 Spruce Street, Philadelphia, PA 19104, USA; yichen.wang@pennmedicine.upenn.edu; 6 Department of Internal Medicine, Roger Williams Medical Center, 825 Chalkstone Ave., Providence, RI 02908, USA; john.miskovsky@chartercare.org; 7 Department of Infectious Disease, Providence VA Medical Center, 830 Chalkstone Ave., Providence, RI 02903, USA; melissa.gaitanis@va.gov; 8 Providence VA Medical Center, Section of Gastroenterology, 830 Chalkstone Ave., Providence, RI 02908, USA; kittichai.promrat@va.gov; 9 Department of Medicine, Division of Cardiology, Warren Alpert School of Medicine, Brown University, 222 Richmond St, Providence, RI 02903, USA; 10 Department of Epidemiology, Brown University School of Public Health, 121 South Main Street, Providence, RI 02903, USA; 11 Center of Innovation for Long Term Services & Support, Veterans Affairs Medical Center, 830 Chalkstone Ave., Providence, RI 02908, USA

**Keywords:** epidemiology, observational study, National Inpatient Sample, non–alcoholic fatty liver disease, NAFLD, non-alcoholic steatohepatitis, NASH, cirrhosis, sepsis, inpatient all–cause mortality

## Abstract

**Background/Objectives:** Patients with Non-Alcoholic Fatty Liver Disease (NAFLD) are reported to have an increased risk of developing severe infections, leading to hospitalizations with sepsis. However, data regarding the impact of comorbid NAFLD on in-hospital outcomes of patients with sepsis is scarce. **Methods:** This nationwide retrospective observational study using discharge data from the National Inpatient Sample (NIS), Healthcare Cost and Utilization Project (HCUP), and Agency for Healthcare Research and Quality included 21,057,911 adult patients who were admitted to hospitals in the United States between 2000 and 2019 with a primary discharge diagnosis of sepsis. These patients were categorized according to the presence or absence of comorbid NAFLD. The twenty-year trend of nationwide NAFLD prevalence among sepsis inpatients was elucidated. Multivariable logistic regression analysis was used to analyze NAFLD’s impact on sepsis outcomes. **Results**: In the twenty-year study period, the prevalence of NALFD among sepsis inpatients trended up from 1.2% in 2000 to 4.2% in 2019. Similar trends were observed in regional analysis. While overall sepsis mortality decreased, comorbid NAFLD in sepsis patients was consistently associated with a higher adjusted in-hospital all-cause mortality rate (adjusted odds ratio (OR), 1.19; 95% confidence interval (CI), 1.07–1.32), higher odds of developing septic shock, and higher likelihood of development of multi–organ dysfunction. **Conclusions**: Comorbid NAFLD in the stage of NASH or cirrhosis is associated with higher in-hospital all-cause mortality and worse clinical outcomes in sepsis inpatients. Addressing this rising epidemic will be of paramount importance to improve sepsis in-hospital outcomes.

## 1. Introduction

Non-Alcoholic Fatty Liver Disease (NAFLD) is the most common chronic liver disease worldwide [1]. NAFLD refers to the presence of hepatic steatosis with no other causes for secondary hepatic fat accumulation (e.g., heavy alcohol consumption, viral hepatitis, etc.) [2]. It is a complex disease spectrum ranging from simple fat buildup in the liver (steatosis) to NASH, which involves inflammation and liver damage, leading to fibrosis or cirrhosis [3]. NAFLD has become an important public health concern not only because of its increasingly high prevalence (prevalence of 10–46% in the United States and 30.69% worldwide) [4], in parallel to the obesity pandemic, but also due to its association with multiple cardiometabolic abnormalities such as metabolic syndrome, type II diabetes mellitus, and cardiovascular disease.

Recently, the increased risk of infection in patients with NAFLD has gained more attention [5]. Chronic low-grade systemic inflammation, which is fundamental in the pathogenesis of NAFLD, is also associated with a higher risk of contracting infection in this patient population [5]. A large body of evidence demonstrates that NAFLD patients are at a higher risk of developing various infections [6,7,8,9], including pneumonia, urinary tract infection (UTI), and COVID-19. Furthermore, NAFLD is associated with worse clinical outcomes in COVID-19 infection and community-acquired pneumonia [10,11]. Notably, a population-based cohort study conducted by Ebrahimi et al. [6], found that patients with biopsy-proven NAFLD were at significantly higher risk of contracting severe infection requiring hospitalization. The mechanism behind this is currently unknown. It may be due to the change in microstructure of liver tissue, leading to impaired immunologic function in the setting of chronic low-grade systemic inflammation [7], such as impaired function of Kupffer cells, dysfunctional neutrophils, and defective innate immunity [5]. A deranged sinusoid microcirculation also led to impaired hepatic microbial clearance, resulting in susceptibility to infection. Moreover, it has also been postulated that the pre-existing proinflammatory state in NAFLD patients may predispose them to cytokine storm, resulting in multi-organ failure in severe COVID-19 infection [12].

Any infection may lead to the development of sepsis, which is defined as life-threatening organ dysfunction caused by a dysregulated host response to infection. Sepsis affects more than 1.7 million adult patients in the United States each year, leading to more than 250,000 deaths annually [13]. Multiple epidemiology studies using administrative data have shown that sepsis incidence has been increasing steadily in the past years. With the global epidemic of NAFLD and the rising incidence of sepsis, it is of paramount importance to elucidate the impact of NAFLD on the outcomes of patients hospitalized with sepsis. This is especially important considering studies demonstrating obesity is associated with decreased mortality in hospitalized patients with sepsis (the so-called obesity paradox) and the shared pathophysiology between NAFLD and obesity [14].

Leveraging a nationwide multi-year dataset of inpatients in the United States, we first studied the temporal trends of NAFLD prevalence among patients hospitalized with sepsis and then elucidated the impact of comorbid NAFLD on the inpatient outcomes of these patients.

## 2. Materials and Methods

### 2.1. Data Collection and Patient Population

The National Inpatient Sample (NIS) dataset from the online Healthcare Cost and Utilization Project Central Distributor (HCUP) was used in the current study. The NIS dataset is the largest publicly available all-payer inpatient discharge database in the United States and consists of weighted data for more than 35 million hospitalizations annually in the United States [15,16]. As detailed in the methodology of HCUP, before 2012, the NIS was created from a sample of hospitals, approximately 20% of a random sample of US community hospitals, not including rehabilitation hospitals. Beginning in 2012, the NIS was redesigned and was generated using a sample of discharges from all community hospitals participating in HCUP, approximating 20% of the random sample of discharges from the US community hospitals, excluding discharges from rehabilitation centers and long-term acute care hospitals. To reflect these sampling design changes, the NIS data element TRENDWT was applied to NIS data from 2000 to 2011 for consistency with those that were used in 2012 and future years of redesigned NIS. Discharges stored in this database were then further stratified by hospital, ownership status, hospital location, census division, hospital teaching status, hospital bed size, etc. Both patient-level and hospital-level trend weights were used to obtain national estimates. Notably, captured encounters represented discharge records, not distinct patients.

Institutional Review Board approval and informed consent were not required for this study because the NIS data were de-identified, and the data are available and accessible to the public through HCUP. The checklist for working with the HCUP dataset was completed [17].

The study population was generated retrospectively by querying the NIS of all hospital admissions of adult patients (ages equal to or more than 18 years old) from 1 January 2000 to 31 December 2019, with a primary discharge diagnosis of sepsis using the International Classification of Diseases, Ninth or Tenth Revision, Clinical Modification (ICD-9-CM or ICD-10 CM) codes (see Supplementary Table S1 for coding details).

### 2.2. Non-Alcoholic Fatty Liver Disease (NAFLD)

The study sample was stratified by the presence or absence of the secondary discharge diagnosis of NAFLD. The discharge diagnosis code of NAFLD is a combination code, which consists of clinical inclusion and exclusion diagnostic criteria for this disease entity, namely with the presence of diagnosis code of fatty liver, non-alcoholic fatty liver, liver steatosis, liver fibrosis/cirrhosis and the exclusion of viral hepatitis, toxoplasma hepatitis, mumps hepatitis, alcoholic fatty liver, alcoholic hepatitis with or without ascites, alcoholic fibrosis and sclerosis of the liver, alcoholic cirrhosis of the liver with or without ascites, alcoholic liver disease, unspecified, alcoholic liver failure, autoimmune hepatitis, Wilson disease, hemochromatosis, lipodystrophy, syphilis of the liver, toxic liver disease, primary sclerosing cholangitis, Budd-Chiari syndrome, clonorchiasis, echinococcus of the liver, fascioliasis, Fabry disease, Gaucher disease, and chronic passive congestion of the liver.

### 2.3. Demographic Information, and Clinical Variables

Demographic information, including patient-level and hospital-level characteristics, such as age, sex, race, hospital region, hospital teaching status, hospital bed size, primary payer, and comorbidities, was collected and examined across the sepsis cohort and then stratified by the presence or absence of NAFLD. The Elixhauser Comorbidity Index was calculated according to the presence or absence of the 31 queried comorbidities (see Supplementary Table S2 for the details of comorbidities included in the Elixhauser Comorbidity Index).

### 2.4. Outcomes

The trends of NAFLD prevalence among sepsis patients were elucidated. Primary outcomes included in-hospital all-cause sepsis mortality. Secondary outcomes included length of stay, in-hospital complications, which included the ratio of developing acute organ dysfunctions (including cardiovascular, hepatic, respiratory, renal, metabolic, neurologic, and hematologic dysfunction), septic shock, rate of acute kidney injury requiring dialysis, being on mechanical ventilation, pressor use, and discharge dispositions (including discharge to home with self-care or home health care, transfer to a short-term hospital, discharge to a skilled nursing facility and other intermediate facility, being left against medical advice, and discharge alive with unknown and missing).

### 2.5. Statistical Analysis

Continuous variables were expressed as mean and standard deviation, while frequencies and percentages were used for categorical variables. Given the extremely large sample size, normality was assumed for the continuous variable, and standardized differences were calculated for baseline characteristics comparisons between the sepsis-NAFLD group and the sepsis-non-NAFLD counterpart. Variable length of stay (LOS) was treated as a count variable. A weighted multivariable logistic model was constructed to evaluate the impact of NAFLD on the adjusted in-hospital all-cause mortality and binary outcomes. The selection of variables included in the initial model was based on univariate screen results (variables with a standardized difference of equal or more than 0.1), clinical consideration, and the previous literature report. After including all the initially selected variables in the model, a stepwise backward selection was undertaken to define the final model. A collinearity check was conducted as well. The final model was adjusted for age, sex, race, insurance status, median household income percentile, hospital region, hospital bed size, hospital teaching status, obesity or BMI, hypertension, hyperlipidemia, metabolic syndrome, congestive heart failure, cardiac arrhythmia, chronic pulmonary disease, diabetes (complicated), renal failure, paralysis, coagulopathy, fluid and electrolytes disorder, tobacco use, alcohol use, and stroke. Multinomial logistic regression was undertaken to compare the discharge dispositions between the groups. Multivariable Poisson regression was undertaken to compare the adjusted LOS between the two groups. Post-estimation predictive margins commands were used to generate the adjusted mortality rate and adjusted LOS in each group [18].

A regional analysis was conducted to elucidate further the trends of NAFLD prevalence and its impact on sepsis mortality based on the four geographic areas of the United States (Northeast, Midwest, South, and West). Stratification analysis was performed stratifying NAFLD based on severity (steatosis, NASH, and cirrhosis), sepsis into sepsis (general), severe sepsis, and septic shock to elucidate the impact of different stages of NAFLD on inpatient outcomes of sepsis with various severity levels. In detail, for the classification of NAFLD, the ICD-10 code K76.0 was used to define steatosis. The subpopulation of comorbid steatosis was generated with the concurrent secondary diagnosis of NAFLD and steatosis; ICD-10 code K75.81 was used to define NASH, and K74.60 for cirrhosis using a similar strategy. For stratification of sepsis, ICD-10 code R65.20 was used to capture severe sepsis, and R65.21 for septic shock (see Supplementary Table S1).

Furthermore, a mediation analysis was undertaken to unravel the mechanism of NAFLD’s impact on sepsis outcome (mortality). The structural equation model was used separately to examine the potential mediating role of metabolic syndrome on the exposure (NAFLD) on the outcomes (sepsis in-hospital all-cause mortality) [19]. The checklist published by the Guideline for Reporting Mediation Analyses of Randomized Trials and Observational Studies (AGReMA) was followed while conducting the mediation analysis (refer to Supplementary Method S1 for details of mediation analysis) [20].

*Sensitivity analysis*: to mitigate the possible effects of severe inflammatory comorbidities or immunological deficient status on sepsis outcomes, a sensitivity analysis was conducted to elucidate the impact of NAFLD on sepsis outcomes after excluding those conditions, such as rheumatoid arthritis, lymphoma, cancer, post-transplant status, chronic end-stage kidney disease, chronic pulmonary disease, congestive heart failure, and stroke.

SAS 9.4 was used to load the data from NIS 2000 to NIS 2004. After loading, the data were then exported to STATA format data. STATA MP17 (Stata Corp., College Station, TX) was used for further analysis. All the other data from NIS2005-2019 were loaded and analyzed with STATA MP17. All *p* values were generated from two-tailed tests, and *p*-value < 0.05 was considered statistically significant.

## 3. Results

### 3.1. Demographics

Sepsis patients with NAFLD were significantly younger, less likely to be black, and more likely to be Hispanic compared to non-NAFLD patients. The sepsis-NAFLD group had a higher ratio of patients with higher comorbidity scores (Elixhauser comorbidity score of more than 4) as compared to the sepsis-non-NAFLD group (79.3% vs. 57.1%), a higher prevalence of obesity (24.5% vs. 12.9%), coagulopathy (32.4% vs. 11.9%), fluids and electrolytes disorder (62.6% vs. 57.2%), and complicated diabetes (20.8% vs. 14.8%), compared to their non-NAFLD counterparts (Table 1).

### 3.2. Outcomes

#### 3.2.1. Temporal Trend of NAFLD Prevalence in Sepsis Patients

**Figure 1:**
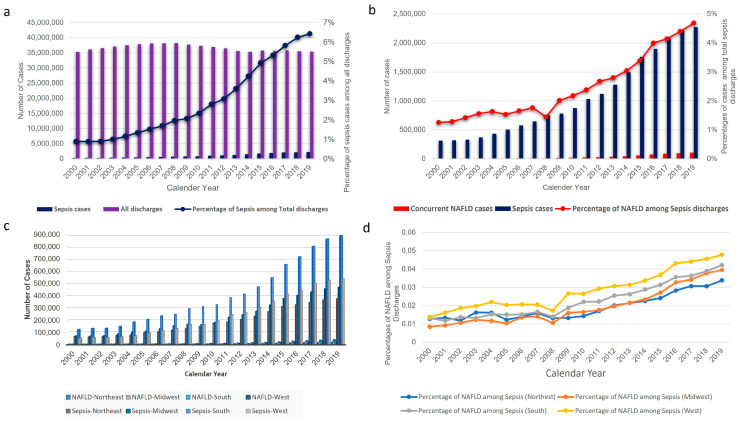
Total discharge numbers, sepsis case numbers, concurrent NAFLD case numbers, and percentages from 2000 to 2019 in the United States, with regional analysis. (**a**) Sepsis cases and the percentage of sepsis cases among total hospital discharges have steadily increased from 311,650 cases, 0.9% in 2000, to 2,274,200 cases, 6.4% among total discharges in 2019. (**b**) The number of sepsis patients with concurrent NAFLD and the percentage of NAFLD among total sepsis admissions trended up steadily, increasing from 3759 cases, 1.2% of sepsis inpatients in the year 2000, to 94,525 cases, 4.2% of sepsis inpatients in the year 2019. (**c**) The number of sepsis patients and concurrent NAFLD case numbers also trended up in the four geographic regions of the United States during the twenty-year study period. (**d**) The percentage of NAFLD among sepsis admissions also trended up steadily in the four geographic regions of the United States, and the West persistently had the highest prevalence of NAFLD among sepsis patients. *NAFLD*—Non-Alcoholic Fatty Liver Disease.

From year 2000 to year 2019, we identified a total of 21,057,911 weighted hospitalizations with a primary discharge diagnosis of sepsis, of which 596,595 (2.8%) had a concurrent secondary discharge diagnosis of NAFLD (see Supplementary Table S3). Notably, during the twenty-year study period, sepsis cases/percentages among total discharges increased (Figure 1a). The concurrent NAFLD case number and prevalence rate among sepsis patients increased remarkably, from 3759 cases, 1.2% of sepsis admissions, in 2000, to 94,525 cases, 4.2% of sepsis admissions, in 2019 (Figure 1b and Supplementary Table S3). Similar trends were observed while studying the prevalence of NAFLD in the four sub geographic areas of the United States (Northeast, Midwest, South, and West) (Figure 1c, d). Notably, the West had a persistently higher prevalence of NAFLD.

#### 3.2.2. Impact of NAFLD on In-Hospital All-Cause Mortality in Sepsis Patients

**Figure 2:**
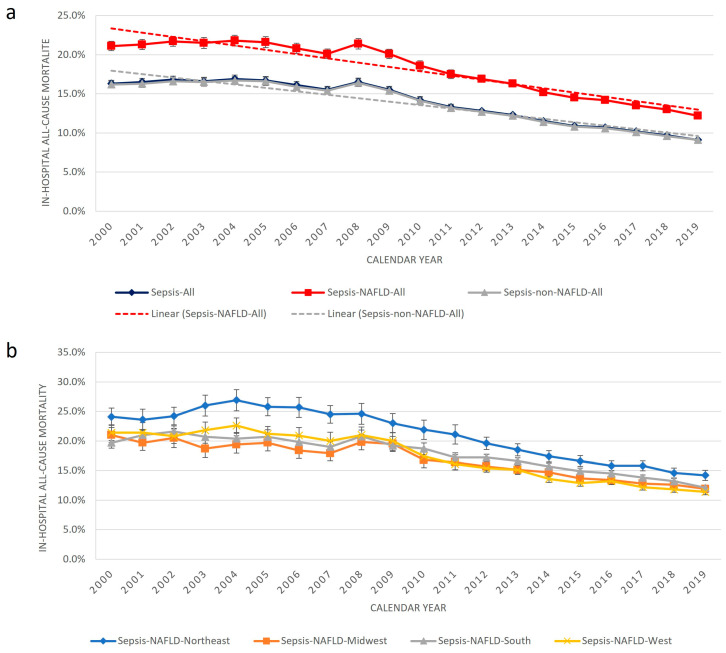
Trend of adjusted in-hospital all-cause mortality rate of sepsis patients, with and without NAFLD, from 2000–2019, in the United States, with regional analysis. (**a**) The in-hospital all-cause mortality for sepsis admissions had trended down steadily, declining from 16.3% in the year 2000 to 9.1% in the year 2019. Similar trends were observed for both the NAFLD group (1.1% to 12.2%) (*p*-value < 0.001 for trend) and non-NAFLD group (16.2% to 9.1%) patients. The mortality rates in the sepsis-NAFLD group patients were persistently higher than those in the non-NAFLD counterparts (*p* < 0.001). (**b**) Similar trends of mortality were also observed in the regional analysis involving the four geographic regions of the United States in NAFLD-sepsis patients (Northeast, Midwest, South, and West), with the Northeast persistently having the highest sepsis mortality rate during the twenty-year study period. *NAFLD*—Non-Alcoholic Fatty Liver Disease.

The all-cause mortality for sepsis admissions has declined, from 16.3% in 2000 to 9.1% in the year 2019. Similar trends were observed in the NAFLD group (21.1% to 12.2%) (*p*-value < 0.001 for trend) and the sepsis-non-NAFLD group. The mortality rates in the sepsis-NAFLD group in each year were consistently higher as compared to the non-NAFLD counterpart (*p* < 0.001) (Figure 2a). Similar trends of sepsis mortality were also observed in regional analysis (Figure 2b). Notably, the Northeast had the highest sepsis mortality rate during the twenty-year study period. The overall all-cause mortality in the sepsis-NAFLD group was 15.1%, which was significantly higher than that in the sepsis-non-NAFLD group (12.4%) (a OR 1.19, 95% CI 1.07–1.32; *p* = 0.001) (Table 2). Please see Supplementary Table S4 for the odds ratio of other variables in the final logistic regression model for mortality.

#### 3.2.3. Impact of NAFLD on the In-Hospital Courses of Sepsis Patients

It was demonstrated in Table 2 that the sepsis-NAFLD group had higher odds of developing cardiovascular complications (aOR 1.14, 95% CI 1.08–1.22, *p* < 0.001), hepatic dysfunction (aOR 5.49, 95% CI 4.96–6.08, *p* < 0.001), hematological dysfunction (aOR 2.2, 95% CI 1.72–2.81, *p* < 0.001), and metabolic dysfunction (aOR 1.27, 95% CI 1.19–1.36, *p* < 0.001). Sepsis-NAFLD group patients were also more likely to develop septic shock (aOR 1.14, 95% CI 1.07–1.22, *p* < 0.001). However, the sepsis-NAFLD group had lower odds of developing respiratory dysfunction (aOR 0.77, 95% CI 0.72–0.82, *p* < 0.001), of being on mechanical ventilation (aOR 0.86, 95% CI = 0.79–0.94, *p* = 0.001), and of developing neurological dysfunction (aOR 0.91, 95% CI 0.85–0.99, *p* = 0.02). There was no significant difference between the two groups regarding the ratio of developing kidney dysfunction. However, the ratio of patients requiring hemodialysis after acute kidney dysfunction was significantly higher in the sepsis-NAFLD (aOR 1.18, 95% CI 1.02–1.37, *p* = 0.031).

**Figure 3:**
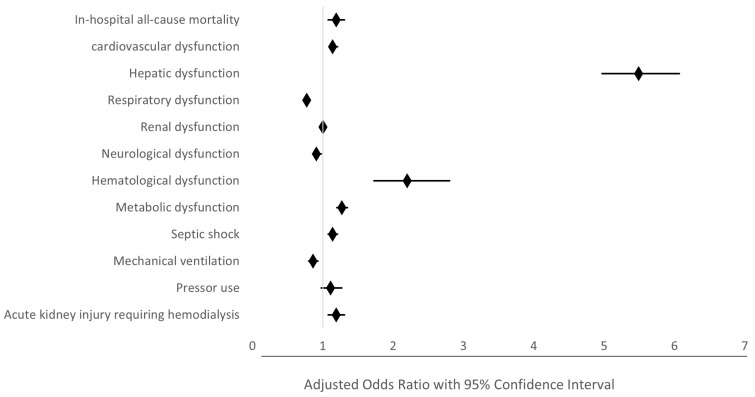
Forrest plot showing clinical outcomes in sepsis admissions with and without NAFLD. Sepsis-NAFLD group patients had higher all-cause mortality and higher odds of developing cardiovascular dysfunction, hepatic dysfunction, renal dysfunction requiring hemodialysis, hematological dysfunction, and metabolic dysfunction. They also had higher odds of developing septic shock as compared to sepsis without NAFLD counterparts. OR and 95% CI were reported for outcome variables. The edges of the diamond point represent the 95% confidence interval limit. The graphical representation in the figure refers to the statistics in Table 2. *NAFLD*—Non-Alcoholic Fatty liver Disease; *OR*—Odds ratio; *CI*—confidence interval.

A forest plot was generated to visualize the impact of NAFLD on the sepsis inpatient outcomes (Figure 3).

#### 3.2.4. Stratification Analysis of the Impact of NAFLD on Sepsis Outcomes Based on NAFLD and Sepsis Severity

It was found that at different stages of NAFLD (steatosis, NASH, and cirrhosis) and sepsis severity (sepsis, severe sepsis, and septic shock), the impact of NAFLD on sepsis outcome varied. Patients with steatosis had lower mortality rates compared to non-steatosis counterparts in different sepsis severity stages (sepsis [aOR0.65, 95% CI 0.53–0.81, *p* < 0.001], severe sepsis [aOR0.55, 95% CI 0.31–0.98, *p* = 0.043], and septic shock [aOR0.70, 95% CI 0.55–0.88], *p* = 0.002) (see Supplementary Table S5). However, in patients with NASH, the mortality rates were significantly higher in patients admitted for sepsis (aOR1.54, 95% CI 1.21–1.95, *p* < 0.001) or septic shock (aOR1.42, 95% CI 1.10–1.83, *p* = 0.008) compared to non-NASH counterparts (see Table 3). In the setting of severe sepsis, there were no significant differences in mortality rates between the two groups. Similar trends were observed in cirrhosis patients with higher mortality rates observed in sepsis or septic shock (Table 4). Regarding the ratio of developing organ dysfunction, the specific types of organ dysfunction developed varied in different stages of NAFLD and sepsis severity stage. However, overall, the results were consistent with the findings before stratification (Table 2, Table 3 and Table 4 and Supplementary Table S5).

#### 3.2.5. Impact of NAFLD on Length of Stay and Discharge Disposition of Sepsis Patients

**Figure 4:**
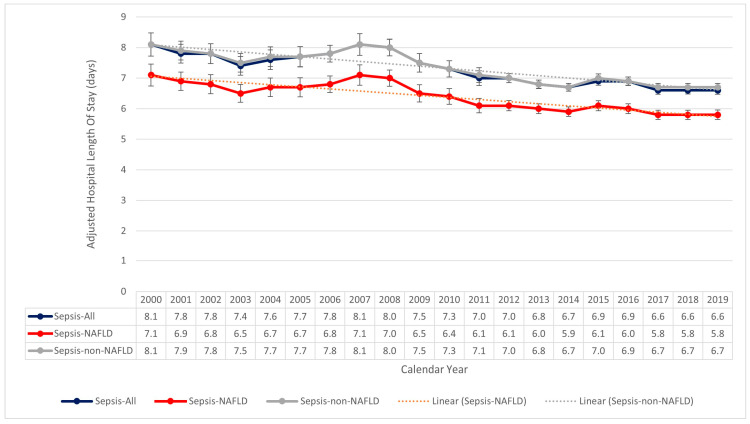
Trends of Adjusted In-Hospital Length of Stay of Sepsis Patients who died in the hospital, with and without NAFLD from 2000 to 2019 in the United States. From the year 2000 to the year 2019, the overall length of stay for sepsis patients who died in the hospital declined from 8.1 days in 2000 to 6.6 days in 2019. Similar trends were observed for both NAFLD (7.1 days to 5.8 days) and non-NAFLD groups (8.1 days to 6.7 days) patients. The length of stay in the sepsis-NAFLD group patients was persistently lower than their non-NAFLD counterparts (*p* < 0.001). *NAFLD*—Non-Alcoholic Fatty Liver Disease.

During the twenty-year study period, the length of stay had declined in all three groups. Among the sepsis patients who were discharged alive, there was no significant difference regarding the length of stay between these two groups in each study year (Supplementary Figure S1). However, among the sepsis patients who died in the hospital, the adjusted overall length of stay in the sepsis-NAFLD group was significantly shorter than that of the sepsis-non-NAFLD group (6.25 days vs. 7.17 days, *p* < 0.001) (Figure 4).

From Supplementary Table S6, among those patients who were discharged alive, overall, 64.8% of sepsis patients were discharged to home/self-care or with home health care, 3.5% of patients were transferred to another short-term hospital, 29.6% of patients were discharged to a skilled nursing facility (SNF) or immediate care facility, about 2.1% of patients left against medical advice, and 0.02% had a missing or unknown discharge disposition. After adjustment, compared to baseline outcomes of being discharged home with self-care or home health care, sepsis-NAFLD patients were more likely to be transferred to a short-term hospital (RRR = 1.24 (95% CI = 1.08–1.42)) and less likely to be discharged to a SNF or intermediate care facility (RRR = 0.86 (95% CI = 0.8–0.93)).

#### 3.2.6. Mediation Analysis

It was found that, statistically, there existed a suppression effect of metabolic syndrome on the impact of NAFLD on sepsis mortality (*p* = 0.043). However, the magnitude of the suppression effect is extremely minimal, with a suppression effect of 0.3% after adjusting for co-variates (see Supplementary Figure S2 and Table S7).

#### 3.2.7. Sensitivity Analysis

After excluding severe inflammatory comorbidities, patients with immunodeficient states, and advanced chronic disease status, it was demonstrated that the aOR for mortality in the sepsis-NAFLD group was 1.28 (95% CI 1.04–1.58, *p* = 0.018) (Supplementary Table S8).

## 4. Discussion

Our current study shows that NAFLD prevalence among sepsis patients increased during the twenty-year study period (2000–2019). While the mortality rate of sepsis inpatients decreased remarkably, sepsis patients with comorbid NAFLD had persistently higher in-hospital mortality rates compared with non-NAFLD counterparts, after adjusting for age, race, gender, BMI, baseline comorbidity burden, insurance status, median household income, and hospital level characteristics. As in our study, Moctezuma-Velázquez et al. found that patients with comorbid NAFLD who were admitted to the hospital for COVID-19 had higher mortality rates as compared to those without NAFLD [21]. Moreover, in line with our current study result, a prospective cohort study conducted by Krznaric et al. also demonstrated that steatotic liver disease might be associated with higher sepsis in-hospital mortality [22]. Our study indicates that comorbid NAFLD might function as an independent negative prognostic factor of inpatient sepsis mortality.

Our regional analysis further confirms the above-observed trends of NAFLD prevalence and sepsis mortality in the four geographic areas of the United States (Northeast, Midwest, South, and West). Our results reveal that the West had the highest NAFLD prevalence, followed by the South, and the Northeast had the highest sepsis mortality, followed by the South. This remarkable geographic variation in NAFLD prevalence and sepsis mortality is consistent with the findings of a study conducted by Gedallovich et al., who conducted a retrospective analysis of claims data from a large, single-payer from 2011 to 2018 that found the West and South to have the highest prevalence of NAFLD with a rising trend [23]. Our current study also echoes the findings from a descriptive analysis of the National Center for Health Statistics demonstrating that the Southeastern United States had the highest sepsis mortality rate [24]. These findings highlight the importance of tailoring the distribution of public health resources while addressing the rising NAFLD epidemic and standardizing the sepsis care management modality nationally.

Furthermore, we found that sepsis-NAFLD inpatients (in the stage of NASH and cirrhosis stage) had a higher rate of developing hepatic dysfunction. This finding is similar to the findings of Huang et al., which demonstrated that patients with NAFLD were more likely to develop liver dysfunction while contracting COVID-19 infection, manifesting as elevated liver enzymes [25]. Their study also noted that severe liver injury and liver failure were not observed in patients with or without NAFLD. However, due to the limitation of the NIS dataset (relying heavily on coding to capture diagnosis), it was difficult to define the severity of liver dysfunction without exact clinical parameters.

Our study also demonstrates that sepsis-NAFLD patients (NASH and cirrhosis stage) had higher odds of developing cardiovascular complications, defined as hypotension or shock. It has been reported that NAFLD is associated with endothelial dysfunction, which is driven by low-grade systemic inflammation [26], and is a risk factor or risk enhancer for atherosclerotic cardiovascular disease, the principal cause of death in patients with NAFLD [27]. However, in the setting of sepsis, this is the first report revealing a possible association between NAFLD and cardiovascular complications. Future studies are warranted to unravel the pathogenesis.

Moreover, the current study reveals that sepsis patients with comorbid NAFLD have a greater risk of developing septic shock compared to those without NAFLD. It is well known that cortisol plays a critical role in counteracting the inflammatory response elicited by sepsis, altering vascular response during sepsis, and thus reducing the risk of septic shock [28]. The hypothalamus-pituitary-adrenal (HPA) axis and response during acute stress situations, including infection, are extremely important to fight infection [29]. There is some human as well as animal evidence that the HPA axis is altered and potentially blunted in the presence of NAFLD. One study of human subjects with NAFLD suggested that there is chronic over-activation of the HPA axis in these patients [30]. However, despite the subtle chronic over-activation of the HPA axis, the corticosterone response to LPS-induced sepsis in rats with NAFLD seemed to be blunted as compared to rats without NAFLD [31], further predicting worse outcomes in the setting of infection, such as developing septic shock. We speculate that there might also exist altered sensitivity to corticosterone due to the baseline endothelial dysfunction in NAFLD in the setting of sepsis. Our results support the abovementioned notion and indicate that NAFLD might function as a predictor for septic shock in sepsis patients.

Patients in the sepsis-NAFLD group had a higher rate of developing acute kidney injury requiring hemodialysis as compared to the non-NAFLD group, indicating that comorbid NAFLD is associated with more severe kidney injury. Like our study, Minhas conducted a nationwide study using NIS [32] and found that NAFLD was associated with an increased risk of acute kidney injury requiring dialysis in patients with a primary diagnosis of heart failure.

Obesity, which is the most common risk factor for NAFLD, was reported to be associated with decreased mortality in sepsis patients in some studies, despite the conclusions being inconclusive [33,34,35]. Meanwhile, one study concluded that sepsis patients with diabetes had a lower mortality rate [36]. Another study indicated that metabolic syndrome was associated with a higher rate of developing ARDS and higher mortality in patients hospitalized with COVID-19 [37].

Our study demonstrates that NAFLD, which bears some common pathophysiologic pathways with obesity, diabetes, and metabolic syndrome, is associated with increased sepsis mortality in the stage of NASH and cirrhosis after adjusting for BMI, diabetes, and metabolic syndrome. One possible pathophysiologic mechanism leading to the increased mortality observed in these patients might be that NAFLD in the stage of NASH or cirrhosis involves the change in the microstructure of the liver tissue, deranged sinusoidal microcirculation, resulting in impaired innate immunity, such as impairment of neutrophils, natural killer (NK) cells, and Kupffer cell functions. Notably, it was reported that hepatic lipid accumulation in NAFLD could promote neutrophil activation and subsequent neutrophil extracellular trap (NET) release [38], which subsequently initiated an additional inflammatory response. On the other hand, the oxidative stress, lipid peroxidation, and generating oxidative stress-derived epitopes after hepatic lipid accumulation would lead to the change in adaptive immune system in NALFD patients, manifesting as stimulating T cells towards differentiation of Th1 pro-inflammatory phenotypes with activation of CD8+ T cells [39]. Furthermore, the baseline excessive pro-inflammatory cytokines, such as TNF-α, IL-6, IL-1α, IL-1β, and IL-18, released after hepatic cell damage in NASH or further stage, might predispose NAFLD patients to cytokine storm in the setting of sepsis, resulting in worse outcomes. In support of our speculation, research conducted by Pisitsak et al. demonstrated that visceral obesity, defined by a high visceral adipose tissue-to-subcutaneous adipose tissue ratio, was associated with adverse outcomes in sepsis patients, probably due to a greater pro- vs. anti-inflammatory response [40]. Furthermore, visceral fat was more strongly associated with fatty liver than subcutaneous fat [41]. Our stratification analysis result, which showed that higher mortality was observed in the NASH subgroup but not in the simple steatosis stage, further indicated liver-injury-related inflammation might be the critical point of NAFLD’s impact on sepsis outcomes. The mediation analysis result indicated NAFLD might also exert its impact on NAFLD through metabolic change from metabolic syndrome (suppression effect). However, given the extremely minimal mediation effect percentage (0.3%), even though the effect is statistically significant, it might not be clinically significant or relevant. We speculate that other unknown pathways are involved in mediating the effect of NAFLD on sepsis outcome. Thus, pathogenesis-wise, here we speculate that NAFLD, which is an indicator of “visceral obesity”, having baseline low-grade systemic inflammation mainly through the liver-related damage in the stage of NASH and cirrhosis, which was different from obesity, promoted a pro-inflammatory response instead of the anti-inflammatory response in the setting of sepsis, with baseline endothelial dysfunction and blunted HPA axis response, along with impaired innate and adaptive immunity, resulting in severe liver injury and cardiovascular complications, which might culminate in septic shock and death. Future research focusing on studying the specific difference between obesity and NAFLD regarding the pro-inflammatory/anti-proinflammatory pathway on a molecular level will not only add the literature on the pathogenesis of sepsis but also shed light on discovering the potential target for treatment to decrease mortality in sepsis-NAFLD patients.

## 5. Conclusions

In conclusion, sepsis patients with comorbid NAFLD in the stage of NASH and cirrhosis had a higher in-hospital all-cause mortality rate and a higher rate of developing septic shock and multi-organ dysfunction as compared to those without NAFLD. NAFLD might function as an independent negative prognostic factor of sepsis inpatient mortality. Addressing this comorbidity (NAFLD) and the underlying pathogenesis of increasing mortality and organ dysfunction would be of paramount importance in decreasing sepsis inpatient mortality. From a public health standpoint, understanding the impact of NALFD on sepsis outcomes may help raise awareness in the population about this prevalent outpatient condition, leading to a more systematic approach to addressing this condition with improved sepsis inpatient outcomes.

## 6. Limitation

Although NIS databases are increasingly used for clinical research, such studies are potentially susceptible to errors related to misclassification and coding inaccuracies. Using administrative data to code for NAFLD might underestimate this clinical condition. In the general population, NAFLD is underdiagnosed, and it was reported that the prevalence of NAFLD in the general population globally was around 30% [4]. Our dataset showed that the prevalence of NAFLD in sepsis patients was 2.8%, which is far below the number reported in the general population. It is highly likely the estimated cases of NAFLD in patients admitted for sepsis heavily underestimated the true burden of the disease in this population. Being that said, there might be cases in the sepsis-non-NAFLD group belonging to the sepsis-NAFLD group resulting in misclassification bias. The true effects of NAFLD on inpatient sepsis outcomes might have been underestimated or overestimated due to the misclassification bias. Furthermore, diagnostic criteria and coding practices have changed in diagnosing NAFLD and sepsis in the past 20 years; this might also affect the consistency of data classification and the observed trends. Besides, the heterogeneity of the study population resulting from evolving diagnosis criteria for NAFLD would also decrease the power of this study. A future validation study linking NIS to hospital records to assess the accuracy of ICD codes in identifying NAFLD and sepsis within the NIS dataset is needed to enhance the credibility of the current study and provide insights about the reliability of using administrative data for future research.

Also, the sepsis study population was generated using the ICD codes; we did not specify the type of sepsis, such as UTI sepsis vs. pneumonia sepsis, which might bias the result as well. Moreover, laboratory parameters such as inflammatory-infectious markers, blood count, and coagulation markers at the presentation that could be predictive of worse clinical outcomes could not be coded and assessed in NIS. Additionally, despite adjustment, there may still be residual confounding factors not accounted for that could influence the relation between NAFLD and sepsis, such as lifestyle factors (diet and physical activity) or genetic predispositions. Moreover, the dataset is confined to inpatient settings, and post-discharge outcomes are not included and inferred. The NIS is currently not linked to any post-discharge outpatient dataset. It is necessary to have data in outpatient post-discharge included to get a complete picture of the impact of NAFLD on sepsis outcomes. This would be an interesting and important direction for future study.

## Figures and Tables

**Table 1:** Baseline demographics and characteristics of patients admitted into the hospital for sepsis with and without NAFLD from 2000 to 2019 in the US *.

Demographic Information	Total (N = 20,604,626)	Sepsis without NAFLD (97.2%, N = 20,023,636)	Sepsis with NAFLD (2.8%, N = 580,990)	Stand. Diff.^†^
**Age, years, mean (SD)**	66.7 (17.2)	67.0 (17.4)	61.7 (14.6)	0.327
**Female sex (%)**	51.6%	51.6%	51.7%	0.583
**Race**				0.291
White	64.4%	64.3%	61.5%	0.058
Black	12.4%	12.5%	9.3%	0.104
Hispanic	8.9%	8.7%	16.4%	0.234
Asian or Pacific Islander, Native Americans, and other	5.7%	5.6%	7.6%	0.081
Missing	8.6%	8.9%	5.2%	0.144
BMI (kg/m^2^) ^‡^				0.395
≤19.9	19.3%	19.8%	8.5%	0.064
20–24.9	11.6%	11.8%	7.3%	0.005
25.0–29.9	8.8%	8.9%	8.3%	0.049
≥30	60.3%	59.6%	75.9%	0.267
**Region**				0.155
Northwest	17.7%	17.7%	14.2%	0.096
Midwest	21.3%	21.2%	18.7%	0.063
South	38.4%	38.7%	39.0%	0.005
West	22.5%	22.4%	28.1%	0.133
**Teaching status**				0.164
Teaching hospital	53.7%	53.5%	61.5%	
Non-teaching hospital	46.3%	46.5%	38.5%	
**Hospital bed size**				0.025
Small	17.0%	17.0%	16.5%	0.020
Medium	28.6%	28.6%	28.1%	0.007
Large	54.4%	54.4%	55.4%	0.023
**Primary payer**				0.253
Medicare	65.2%	65.5%	53.4%	0.250
Medicaid	11.7%	11.6%	16.6%	0.146
Private	17.2%	17.0%	22.9%	0.149
Self-pay	3.5%	3.5%	4.1%	0.031
No charge and other	2.4%	2.4%	3.0%	0.039
**Comorbidity**				
Obesity	13.3%	12.9%	24.5%	0.3
Congestive heart failure	24.6%	24.7%	23.1%	0.038
Renal failure	23.8%	23.7%	26.3%	0.06
Paralysis	3.3%	3.3%	1.6%	0.108
Chronic pulmonary disease	27.9%	28.0%	25.7%	0.05
Metastatic cancer	4.8%	4.9%	4.0%	0.045
Coagulopathy	12.5%	11.9%	32.4%	0.509
Cardiac Arrhythmia	28.3%	28.5%	24.5%	0.089
Valvular disease	6.9%	6.9%	7.4%	0.02
Pulmonary circulation disorder	5.2%	5.2%	6.6%	0.061
Other neurological disorder	20.6%	20.7%	18.9%	0.044
Periphery artery disease	8.1%	8.2%	7.3%	0.030
Liver disease	8.6%	6.1%	95.5%	4.01
Weight loss	15.4%	15.3%	15.9%	0.015
Fluid and electrolyte disorder	57.3%	57.2%	62.6%	0.112
Stroke	7.6%	7.6%	5.4%	0.091
Solid organ transplant	0.9%	0.9%	1.1%	0.014
Tabacco	25.0%	24.9%	27.2%	0.054
HLD	27.1%	27.1%	26.5%	0.013
HTN uncomplicated	33.7%	33.7%	32.1%	0.034
HTN complicated	23.9%	23.8%	25.9%	0.047
Diabetes uncomplicated	19.7%	19.6%	23.6%	0.097
Diabetes complicated	15%	14.8%	21.8%	0.182
Metabolic syndrome	0.1%	0.1%	0.4%	0.013
Hypothyroidism	13.1%	13.0%	14.4%	0.041
Peptic ulcer disease	0.8%	0.8%	1.2%	0.038
AIDS/HIV	0.6%	0.6%	1.1%	0.058
Lymphoma	2.0%	2.0%	1.7%	0.018
Solid tumor without metastasis	7.6%	7.6%	8.3%	0.025
Rheumatoid arthritis	4.1%	4.1%	4.4%	0.014
Blood loss anemia	1.1%	1.1%	1.5%	0.031
Deficiency anemia	5.0%	5.0%	6.3%	0.058
Psychosis	2.3%	2.3%	1.9%	0.031
Depression	12.1%	12.1%	12.9%	0.026
Alcohol abuse	4.8%	4.8%	0	0.315
Drug abuse	4.5%	4.5%	6.0%	0.071
**Elixhauser Comorbidity Index**				0.59
0–1	10.7%	11.0%	2.1%	0.365
2–3	31.6%	32.0%	18.7%	0.311
4–5	32.8%	32.8%	33.9%	0.025
≥6	24.9%	24.3%	45.4%	0.456

*NAFLD*—Non-Alcoholic Fatty Liver Disease; *US*—United States; *Stand. Diff.*—standardized difference; BMI—Body Mass Index; kg/m^2^, kilogram per square meter; *HLD*—hyperlipidemia; *HTN*—hypertension; *AIDS*—acquired immune deficiency syndrome; *HIV*—human immunodeficiency virus. * Data source: National Inpatient Sample—(NIS), 2000–2014, and 2015 quarter 1-quarter 3 (2015 Q1–Q3), 2016–2019 (except for BMI, see below); ^†^—“Standardized difference” displays the difference in means or proportions divided by standard error; the absolute value of a standardized difference of more than 0.1 was interpreted as an imbalance between the groups in the current study. ^‡^—Data source generating the BMI parameter was from NIS 2005–2014, 2015 quarter 1-quarter 3 (2015 Q1–Q3), 2016–2019.

**Table 2:** In-hospital clinical outcomes of patients admitted for sepsis with and without NAFLD.

Outcomes	Total	Univariate Results		Unadjusted OR (95% CI)	Adjusted OR (95% CI) *	*p*-Value
		Sepsis-Non-NAFLD	Sepsis-NAFLD			
**Died**	12.5%	12.4%	15.1%	1.25 (1.23–1.28)	1.19 (1.07–1.32)	0.001
**Type of Organ** **dysfunction**						
**Cardiovascular**	25.2%	25.0%	30.6%	1.32 (1.28–1.37)	1.14 (1.08–1.22)	<0.001
**Hepatic**	3.4%	2.9%	14.4%	5.65 (5.38–5.92)	5.49 (4.96–6.08)	<0.001
**Respiratory**	34.2%	34.4%	30.7%	0.84 (0.82–0.87)	0.77 (0.72–0.82)	<0.001
**Renal**	39.8%	39.6%	43.7%	1.18 (1.14–1.22)	1.002 (0.94–1.06)	0.925
**Neurological**	20.0%	20.0%	19.3%	0.96 (0.92–1.00)	0.91 (0.85–0.99)	0.02
**Hematologic**	12.0%	11.4%	27.9%	3.02 (2.92–3.13)	2.20 (1.72–2.81)	<0.001
**Metabolic**	24.8%	24.5%	31.9%	1.45 (1.40–1.50)	1.27 (1.19–1.36)	<0.001
**Septic shock**	19.8%	19.6%	24.8%	1.35 (1.31–1.40)	1.14 (1.07–1.22)	<0.001
**Procedures**						
**Mechanical ventilation**	12.2%	12.2%	13.2%	1.10 (1.05–1.15)	0.86 (0.79–0.94)	0.001
**Acute kidney injury** **requiring hemodialysis**	5.7%	5.6%	8.5%	1.58 (1.46–1.72)	1.18 (1.02–1.37)	0.031
**Pressor use**	3.9%	3.9%	5.4%	1.41 (1.31–1.52)	1.11 (0.97–1.28)	0.12

*NAFLD*—Non-Alcoholic Fatty Liver Disease; %, percentage; *OR*—Odds Ratio; *CI*—confidence interval. * The results were adjusted for age, sex, race, median house income percentile, insurance status, hospital region, hospital bed size, hospital teaching status, BMI, congestive heart failure, cardiac arrhythmia, chronic pulmonary disease, diabetes (complicated), renal failure, hypertension, hyperlipidemia, metabolic syndrome, paralysis, coagulopathy, fluid and electrolyte disorder, smoking, alcohol use, and stroke.

**Table 3:** Stratification analysis: in-hospital outcomes of patients admitted for sepsis, severe sepsis, and septic shock with and without NASH.

NAFLD Stage	Outcomes	Sepsis		Severe Sepsis		Septic Shock	
		Adjusted OR (95% CI) *	*p* Value	Adjusted OR (95% CI)	*p* Value	Adjusted OR (95% CI)	*p* Value
NASH	Died	1.54 (1.21–1.95)	<0.001	0.93 (0.42–2.02)	0.848	1.42 (1.10–1.83)	0.008
	Type of organ dysfunction						
	Hepatic	8.76 (7.21–10.66)	<0.001	10.89 (7.12–16.64)	<0.001	4.62 (3.60–9.52)	<0.001
	Respiratory	0.75 (0.64–0.88)	0.001	0.62 (0.43–0.89)	0.01	0.92 (0.72–1.18)	0.519
	Cardiovascular	1.31 (1.13–1.52)	<0.001	0.83 (0.49–1.41)	0.491	1	–
	Renal	0.94 (0.81–1.10)	0.463	0.73 (0.53–1.001)	0.051	1.16 (0.89–1.53)	0.275
	Neurological	0.98 (0.81–1.18)	0.801	0.88 (0.60–1.29)	0.507	0.80 (0.63–1.02)	0.07
	Hematologic	2.78 (1.67–4.62)	<0.001	3.65 (1.11–12.05)	0.033	4.39 (1.38–13.97)	0.012
	Metabolic	1.44 (1.22–1.71)	<0.001	1.20 (0.83–1.73)	0.324	1.54 (1.17–2.01)	0.002
	Septic shock	1.27 (1.08–1.50)	0.004	1.46 (0.35–6.02)	0.604	–	–
	Procedures						
	Mechanical Ventilation	0.97 (0.79–1.20)	0.80	0.61 (0.34–1.11)	0.104	0.99 (0.78–1.27)	0.952
	Acute kidney injury requiring hemodialysis	1.05 (0.76–1.45)	0.776	1.72 (0.81–3.62)	0.156	1.08 (0.79–1.18)	0.636
	Pressor use	0.96 (0.68–1.35)	0.804	1.26 (0.39–4.01)	0.701	0.95 (0.69–1.32)	0.763

*NASH*—Non-alcoholic steatohepatitis; *NAFLD*—Non-Alcoholic Fatty Liver Disease; *OR*—Odds Ratio; *CI*—confidence interval. * The results were adjusted for age, sex, race, median house income percentile, insurance status, hospital region, hospital bed size, hospital teaching status, BMI, congestive heart failure, cardiac arrhythmia, chronic pulmonary disease, diabetes (complicated), renal failure, hypertension, hyperlipidemia, metabolic syndrome, paralysis, coagulopathy, fluid and electrolytes disorder, smoking, alcohol use, and stroke.

**Table 4:** Stratification analysis: in-hospital outcomes of patients admitted for sepsis, severe sepsis, and septic shock with and without cirrhosis.

NAFLD Stage	Outcomes	Sepsis		Severe Sepsis		Septic Shock	
		Adjusted OR (95% CI) *	*p* Value	Adjusted OR (95% CI)	*p* Value	Adjusted OR (95% CI)	*p* Value
Cirrhosis	Died	1.46 (1.27–1.69)	<0.001	0.94 (0.61–1.42)	0.758	1.47 (1.26–1.71)	<0.001
	Type of organ dysfunction						
	Hepatic	7.98 (7.01–9.08)	<0.001	10.92 (8.23–14.47)	<0.001	4.02 (3.39–4.75)	<0.001
	Respiratory	0.74 (0.67–0.82)	<0.001	0.62 (0.50–0.77)	<0.001	0.82 (0.71–0.96)	0.012
	Cardiovascular	1.29 (1.17–1.42)	<0.001	0.98 (0.72–1.32)	0.877	0.24 (0.03–1.84)	0.168
	Renal	0.9890.89–1.08)	0.644	0.88 (0.73–1.07)	0.216	0.89 (0.76–1.04)	0.129
	Neurological	1.11 (0.99–1.24)	0.070	1.21 (0.97–1.50)	0.084	0.87 (0.75–1.01)	0.069
	Hematologic	3.37 (2.35–4.84)	<0.001	4.75 (2.02–11.15)	<0.001	1.95 (1.12–3.40)	0.019
	Metabolic	1.33 (1.19–1.48)	<0.001	1.20 (0.97–1.50)	0.094	1.03 (0.88–1.22)	0.689
	Septic shock	1.27 (1.14–1.41)	<0.001	0.47 (0.11–1.91)	0.289	–	–
	Procedures						
	Mechanical ventilation	0.91 (0.79–1.04)	0.177	0.50 (0.34–0.74)	<0.001	0.93 (0.80–1.08)	0.349
	Acute kidney injury requiring hemodialysis	1.18 (0.95–1.47)	0.139	1.12 (0.64–1.96)	0.687	1.09 (0.88–1.36)	0.417
	Pressor use	1.24 (1.02–1.50)	0.034	0.89 (0.35–2.26)	0.804	1.11 (0.92–1.34)	0.273

*NAFLD*—Non-Alcoholic Fatty Liver Disease; *OR*—Odds Ratio; *CI*—confidence interval. * The results were adjusted for age, sex, race, median house income percentile, insurance status, hospital region, hospital bed size, hospital teaching status, BMI, congestive heart failure, cardiac arrhythmia, chronic pulmonary disease, diabetes (complicated), renal failure, hypertension, hyperlipidemia, metabolic syndrome, paralysis, coagulopathy, fluid and electrolytes disorder, smoking, alcohol use, and stroke.

## Data Availability

All data are available on the HCUP website: https://hcup-us.ahrq.gov/nisoverview.jsp.

## Supplementary Materials

The following supporting information can be downloaded at: https://www.mdpi.com/article/10.3390/jcm13195737/s1. Method S1: Mediation path analysis methodology; Table S1: ICD-9-CM and ICD-10-CM diagnoses and procedure codes for all variables; Table S2: Elixhauser Comorbidity Index variable; Table S3: Case number of total sepsis discharges, concurrent NAFLD cause numbers, and percentage of NAFLD among sepsis discharges from 2000 to 2019 in the United States; Table S4: Results of the multivariable logistic regression model assessing the impact of NAFLD on sepsis inpatient all-cause mortality; Table S5: Stratification analysis: In-hospital outcomes of patients admitted for sepsis, severe sepsis, and septic shock with and without steatosis; Table S6: Discharge disposition of patients admitted for sepsis with and without NAFLD groups; Table S7: Mediation analysis of the role of metabolic syndrome on the impact of NAFLD on sepsis inpatient mortality; Table S8: Sensitivity analysis: Results of the multivariable logistic regression model assessing the impact of NAFLD on sepsis inpatient all-cause mortality after excluding severe inflammatory comorbidities, advanced cardiovascular disease, and chronic kidney failure; Figure S1: Trends of adjusted in-hospital length of stay of sepsis patients who were discharged alive, with and without NAFLD, from 2000 to 2019 in the United States; Figure S2: Mediation analysis of metabolic syndrome on the impact of NAFLD on sepsis in-hospital mortality.
