# Supplementary material for: Impact of Non-Alcoholic Fatty Liver Disease on Sepsis Inpatient Outcomes: A Nationwide Sample Analysis (2000–2019)
Source: J Clin Med. 2024 Sep 26;13(19):5737. doi: 10.3390/jcm13195737 (PMC11476451; doi:10.3390/jcm13195737)
Supplement: Supplementary file 1 [file jcm-13-05737-s001.zip › jcm-3190748-supplementary.pdf]

Supplementary information

Title: Impact of Non-Alcoholic Fatty Liver Disease on Sepsis Inpatient Outcomes: A Nationwide Sample Analysis (2000-2019)

Authors:

Xiuhong Lyu MD, PhD[1,2]; Bolun Liu MD[3]; Yiting Li MD[4]; Yichen Wang MD[5]; John Miskovsky MD[6]; Melissa Gaitanis MD[7]; Kittichai Promrat MD[8]; Wen-Chih Wu MD, MPH[9, &].

Affiliations

[1] Department of Epidemiology, School of Public Health, Brown University, 121 South Main Street, Providence, RI 02903, USA.

[2] Department of Adult Medicine, Brockton Neighborhood Health Center, 63 Main Street, Brockton, MA 02301, USA

[3] Department of Hospital Internal Medicine, Mayo Clinic Health System, 1025 Marsh Street, Mankato, MN 56001, USA.

[4] Division of Gastroenterology and Hepatology, 2500 Marble Ave, University of New -Mexico Health Science Center, Albuquerque, NM 87106, USA.

[5] Division of Hospital Medicine, the Hospital of the University of Pennsylvania and Penn Presbyterian Medical Center, 3400 Spruce Street, Philadelphia, PA19104, USA.

[6] Department of Internal Medicine, Roger Williams Medical Center, 825 Chalkstone Ave, Providence, RI 02908, USA.

[7] Department of Infectious Disease, Providence VA Medical Center, 830 Chalkstone Ave, Providence, RI 02903, USA.

[8] Providence VA Medical Center, Section of Gastroenterology, 830 Chalkstone Ave, Providence, RI 02908, USA.

[9] Department of Medicine, Division of Cardiology, Warren Alpert School of Medicine, Brown University; Department of Epidemiology, Brown University School of Public Health; Center of Innovation for Long Term Services & Support, Veterans Affairs Medical Center, 830 Chalkstone Ave, Providence, RI 02908, USA.

[&] **Corresponding author**:
Name: Wen-Chih Wu, Professor of Medicine & Epidemiology.
Address: Department of Medicine, Division of Cardiology, Warren Alpert School of Medicine, Brown University; Department of Epidemiology, Brown University School of Public Health; Center of Innovation for Long Term Services & Support, Veterans Affairs Medical Center, 830 Chalkstone Ave, Providence, RI 02908, USA.
Electronic Email address: wen-chih_wu@brown.edu

Table of contents

Supplementary Method S1:

Mediation path analysis methodology

Supplementary Table S1:

ICD-9-CM and ICD-10-CM diagnoses and procedure codes for all Variables.

Supplementary Table S2:

Elixhauser Comorbidity Index variable

Supplementary Table S3:

Case number of total sepsis discharges, concurrent NAFLD cause numbers, and percentage of NAFLD among sepsis discharges from 2000 to 2019 in the US.

Supplementary Table S4:

Results of the multivariate logistic regression model assessing the impact of NAFLD on sepsis inpatient all-cause mortality.

Supplementary Table S5:

Stratification analysis: In-hospital outcomes of patients admitted for Sepsis, severe Sepsis, Septic shock with and without steatosis

Supplementary Table S6:

Discharge disposition of patients admitted for sepsis with and without NAFLD groups.

Supplementary Table S7:

Mediation analysis of the role of metabolic syndrome on the impact of NAFLD on sepsis inpatient mortality.

Supplementary Table S8:

Sensitivity analysis: Results of the multivariable logistic regression model assessing the impact of NAFLD on sepsis inpatient all-cause mortality after excluding severe inflammatory comorbidities, advanced cardiovascular disease, and chronic kidney failure.

Supplementary Figure S1:

Trends of Adjusted In-Hospital Length of Stay of Sepsis Patients who were discharged alive, With and Without NAFLD from 2000 to 2019 in the US

Supplementary Figure S2:

Mediation analysis of metabolic syndrome on the impact of NAFLD on sepsis in-hospital mortality.

Supplementary Method S1: Mediation path analysis methodology

The structural equation model was used separately to examine the potential mediating role of metabolic syndrome on the exposure (NAFLD) on the outcomes (sepsis in-hospital all-cause mortality). The checklist published by the Guideline for Reporting Mediation Analyses of Randomized Trials and Observational Studies (AGReMA) was followed while conducting the mediation analysis.[1] The analysis was adjusted for age, sex, race, insurance status, hospital bed size, hospital teaching status, obesity, congestive heart failure, cardiac arrhythmia, chronic pulmonary disease, diabetes(complicated), renal failure, paralysis, coagulopathy, fluid and electrolyte disorder, alcohol use, stroke, HTN, and HLD. Metabolic syndrome is the mediator. There were three equations per regression analysis in the path models. Equation a): the association between NAFLD (predictor) and metabolic syndrome (mediator), adjusting for the co-variables mentioned above; Equation b): the association between metabolic syndrome and sepsis inpatient mortality(outcome), adjusting for the co-variables as mentioned above. Equation c): the association between NAFLD and sepsis inpatient mortality (total effects), and Equation c') (direct effects): the association between NAFLD and sepsis inpatient mortality, accounting for the mediating role of metabolic syndrome. The percentage of mediating roles was calculated as the ratio of difference between equation c' and equation c. Mediation effect is confirmed under the premise that: there were significant associations between predictor and mediator, mediator and outcome, predictor and outcome, and the association between predictor and outcome attenuated after including the mediator.[2] On the other hand, if the magnitude of the association between the predictor and outcome increased after including the mediator, then there is suppression.[2] The mediation analysis was carried out using the STATA 17 MP *medsem* package. Please see below the results of the mediation analysis. A statistically significant mediation or suppression percentage of less than 1% was considered to be minimal, and 1% or larger was deemed to be partial.

Reference

1. Lee H, Cashin AG, Lamb SE, Hopewell S, Vansteelandt S, VanderWeele TJ, MacKinnon DP, Mansell G, Collins GS, Golub RM *et al*: **A Guideline for Reporting Mediation Analyses of Randomized Trials and Observational Studies: The AGReMA Statement**. *JAMA* 2021, **326**(11):1045-1056.
2. MacKinnon DP, Krull JL, Lockwood CM: **Equivalence of the mediation, confounding and suppression effect**. *Prev Sci* 2000, **1**(4):173-181.

Supplementary Table S1: ICD-9-CM and ICD-10-CM diagnoses and procedure codes for all variables[*]

Diagnoses		ICD-9-CM diagnoses or procedure codes[a]	ICD-10-CM diagnoses or procedure codes[b]
**Sepsis**		995.91, 995.92, 785.52, 38.0, 038.1x, 038.2, 038.3, 038.4x, 038.8, 038.9, 790.7, 117.9, 112.5, 112.81, 115.04, 115.14, 115.94, 112.83, 003.1, 003.21, 036.2, 036.3, 036.0, 036.1, 036.42, 020.2, 022.3, 098.89, 098.84, 098.82	R65.20, R65.21, A02.1, A22.7, A26.7, A32.7, A40.0, A40.1, A40.3, A40.8, A40.9, A41.01, A41.02, A41.1, A41.2, A41.3, A41.4, A41.50, A41.51, A41.52, A41.53, A41.59, A41.81, A41.89, A42.7, A54.86, B37.7, A41.9, A20.0, R78.81, B38.7, B37.6, B39.4 & I39, B39.5 & I39, B39.9 & I39, B37.5, A02.21, A39.4, A39.1, A39.0, A39.81, A39.51, A20.7, A54.86, A54.83, A54.81
**NAFLD**	**Inclusion codes**	571.8, 571.9, 571.5	K76.0, K75.81, K74.1, K76.9, K74.0, K74.2, K74.6, K74.60, K74.69, K75.89, K75.9
	**Exclusion of other etiologies**		
	Viral hepatitis	070.0, 071.1, 070.21, 070.31, 070.20, 070.30, 070.52, 070.51, 070.41, 070.53, 070.49, 070.59, 070.9, 070.33, 070.32, 070.54, 070.6, 070.70, 070.71, 070.44, V02.62, 573.1	B15.0, B15.9, B16.0, B16.1, B16.2, B16.9, B17.0, B17.10, B17.11, B17.2, B17.8, B17.9, B18.0, B18.1, B18.2, B18.8, B19.0, B19.10, B19.11, B19.20, B19.21, B19.9, B25.1
	Toxoplasma hepatitis	130.5	B58.1
	Mumps hepatitis	072.71	B26.81
	Alcoholic fatty liver	571.0	K70.0
	Alcoholic hepatitis without ascites/with ascites	571.1	K70.10, K70.11
	Alcoholic fibrosis and sclerosis of the liver	571.2	K70.2
	Alcoholic cirrhosis of the liver without ascites/with ascites	571.2	K70.30, K70.31
	Alcoholic liver disease, unspecified	571.3	K70.9
	Alcoholic hepatic failure	571.3	K70.4, K70.40, K70.41

	Autoimmune hepatitis	571.42, 571.49	K75.4
	Wilson Disease	275.1	E83.01
	hemochromatosis	275.01, 275.02, 275.03, 275.09, 275.0	E83.11
	Lipodystrophy	272.6	E88.1
	Toxic liver disease	573.3, 573.8	K71.x (K71.0, K71.1, K71.2, K71.3, K71.4, K71.5, K71.6, K71.7, K71.8, K71.9)
	Syphilis of the liver	095.3	A52.74
	Primary sclerosing cholangitis	576.1	K83.01
	Primary biliary cholangitis	571.6	K74.3, K74.5
	Budd-Chiari syndrome	453.0	I82.0
	Clonorchiasis	121.1	B661
	Echinococcus of liver	122.0, 122.5, 122.8	B67.0, B67.5, B67.8
	Fascioliasis	121.3	B66.3
	Fabry disease	272.7	E75.21
	Gaucher disease	N/A	E75.22, E77.0, E77.1
	Chronic passive congestion of the liver	573.0	K76.1
	Opisthorchiasis	121.0	B66.0
Steatosis			K76.0
NASH			K75.81
Cirrhosis			K74.60
**Comorbidities**			
Obesity		278, 278.0, 278.00, 278.01, 278.03, V85.3, V85.30, V85.31, V85.32, V85.33, V85.34, V85.35, V85.36, V85.37, V85.38, V85.39, V85.4, V85.41, V85.42, V85.43, V85.44, V85.45	E66.0, E66.8, E66.9, E66.1, E66.01, E66.09, E66.2, Z68.30, Z68.31, Z68.32, Z68.33, Z68.34, Z68.35, Z68.36, Z68.37, Z68.38, Z68.39, Z68.41, Z68.42, Z68.43, Z68.44, Z68.45
HTN (complicated, uncomplicated)		Elixhauser comorbidity coding	Elixhauser comorbidity coding
hyperlipidemia		272.0,272.1,272.2,272.3, 272.4, 272.5,272.8,272.9	E78.x (E78.00, E78.01, E78.1, E78.2, E78.4, E78.5, E78.6, E78.81, E78.89, E78.9, E78.3
Diabetes mellitus (complicated, uncomplicated)		Elixhauser comorbidity coding	Elixhauser comorbidity coding
Renal failure		Elixhauser comorbidity coding	Elixhauser comorbidity coding
Chronic congestive heart failure		Elixhauser comorbidity coding	Elixhauser comorbidity coding
Paralysis		Elixhauser comorbidity coding	Elixhauser comorbidity coding
Chronic pulmonary disease		Elixhauser comorbidity coding	Elixhauser comorbidity coding
Metastatic cancer		Elixhauser comorbidity coding	Elixhauser comorbidity coding
Coagulopathy		Elixhauser comorbidity coding	Elixhauser comorbidity coding
Cardiac Arrhythmia		Elixhauser comorbidity coding	Elixhauser comorbidity coding

Valvular disease	Elixhauser comorbidity coding	Elixhauser comorbidity coding
Pulmonary circulation disorder	Elixhauser comorbidity coding	Elixhauser comorbidity coding
Other neurological disorder	Elixhauser comorbidity coding	Elixhauser comorbidity coding
Periphery artery disease	Elixhauser comorbidity coding	Elixhauser comorbidity coding
Liver disease	Elixhauser comorbidity coding	Elixhauser comorbidity coding
Weight loss	Elixhauser comorbidity coding	Elixhauser comorbidity coding
Fluid and electrolyte disorder	Elixhauser comorbidity coding	Elixhauser comorbidity coding
Stroke, systemic thromboembolism, TIA	V12.54, V12.59, 362.30, 444.21.444.22, 444.81, 444.89, 444.0, 444.01, 444.09, 444.1, 433.91, 434.01, 434.91, 435.0, 435.1, 435.3, 435.8, 362.34, 437.7, 435.2, 435.9, 438.9, 438.0, 438.12, 438. 13, 438.14, 438.19	I69.3, I69.30, I69.31, I69.32, I69.33, I69.34, I69.35, I69.39, I69.9, I69.310, I69.311, I69.312, I69.313, I69.314, I69.315, I69.318, I69.319, I69.329, I69.321, H34.0, H34.1, H34.2, H34.9, Z86.73, I63.x (I63.0, I63.1, I63.2, I63.3, I63.4, I63.5, I63.6, I63.8, I63.9, I63.00, I63.01), I74.x (I74.0, I74.1, I72.2, I74.3, I74.4, I74.5, I74.8, I74.01, I74.09, I74.10, I74.11, I74.19), G45.x(G45, G45.0, G45.1, G45.2, G45.3, G45.4, G45.8, G45.9) I69.923, I69.922, I69.928
Peptic ulcer disease	Elixhauser comorbidity coding	Elixhauser comorbidity coding
AIDS/HIV	Elixhauser comorbidity coding	Elixhauser comorbidity coding
Lymphoma	Elixhauser comorbidity coding	Elixhauser comorbidity coding
Solid tumor without metastasis	Elixhauser comorbidity coding	Elixhauser comorbidity coding
Rheumatoid arthritis	Elixhauser comorbidity coding	Elixhauser comorbidity coding
Blood loss anemia	Elixhauser comorbidity coding	Elixhauser comorbidity coding
Deficiency anemia	Elixhauser comorbidity coding	Elixhauser comorbidity coding
Psychosis	Elixhauser comorbidity coding	Elixhauser comorbidity coding
Depression	Elixhauser comorbidity coding	Elixhauser comorbidity coding
Alcohol abuse	Elixhauser comorbidity coding	Elixhauser comorbidity coding
Drug abuse	Elixhauser comorbidity coding	Elixhauser comorbidity coding
hypothyroidism	Elixhauser comorbidity coding	Elixhauser comorbidity coding
Solid organ transplant	V42.1, V42.6, V42.7, V42.84, V42.0, V42.83. V42.89	Z94.1, Z94.2, Z94.4, Z94.82, Z94.0, Z94.83, Z94.89
Tobacco use	305.1, V15. 82, 649.0x	Z87.891, F17.x
Metabolic syndrome	277.8, 277.7	E88.81
BMI <=19.9 (kg/m2)	V85.0	Z68.1
BMI 20-24.9 (kg/m2)	V85.1	Z68.20, Z68.21, Z68.22, Z68.23, Z68.24,
BMI 25-29.9 (kg/m2)	V85.2, V85.21, V85.22, V85.23, V85.24, V85.25	Z68.25, Z68.26, Z68.27, Z68.28, Z68.29
BMI 30-34.9 (kg/m2)	V85.3 V85.30, V85.31, V85.32, V85.33, V85.34	Z68.30, Z68.31, Z68.32, Z68.33, Z68.34,
BMI 35-39.9 (kg/m2)	V85.35, V85.36, V85.37, V85.38, V85.39	Z68.35, Z68.36, Z68.37, Z68.38, Z68.39
BMI Over 40 (kg/m2)	V85.41, V85.42, V85.43, V85.44, V84.45.	Z68.41, Z68.42, Z68.43, Z68.44, Z68.45
**In-hospital complications**		

**Organ dysfunction**	Cardiovascular	785.5, 785.50, 785.59, 785.51, 785.52, 458.8, 458.9, 796.3	R57.9, R57.1, R57.8, R57.0, R65.21, I95.89, I95.9, R03.1, T81.11XA
	Renal	583.6, 583.7, 584.6, 584.7, 584.5, 584.8,584.9	N17.0, N17.1, N17.2, N17.8, N17.9
	Hepatic	570, 572.2, 573.3, 573.4	K72.0, K72.00, K72.01, K72.90, K72.91, K76.82, K76.3
	Respiratory	518.81, 518.82, 518.84, 518.53, 518.51, 518.85, 786.09, 799.1	J96.01, J96.20, J95.822, J95.821, J96.2, J96.21, J96.22, J96.02, J96.00, J96.20, J96.90, J96.91, J96.92, J80, R06.03, R09.2
	Hematologic	286.6, 286.7, 286.9, 287.4, 287.41, 287.49, 287.5	D65, D68.32, D68.4, D68.8, D68.9, D69.6, D69.59, D69.51
	Metabolic	276.2	E87.2
	Neurologic	293, 293.0, 293.1, 293.8, 293.81, 293.82, 293.83, 293.84, 293.89, 293.9, 348.1, 348.3, 348.30, 348.31, 348.39, 780.01, 780.09, Procedure code 89.14	F05, F06.2, F06.0, F06.30, F06.4, F06.1, F53, F06.8, G93.1, G93.40, G93.41, G93.49, I67.83, R40.20, R40.0, R40.1 Procedure code: 4A0034Z, 4A0074Z, 4A0084Z, 4A00X4Z, 4A1034Z, 4A1074Z, 4A1084Z, 4A10X4Z
**In hospital procedures and others**	Septic shock	785.59, 785.52	R65.21
	Acute kidney injury requires hemodialysis	583.6, 583.7, 584.6, 584.7, 584.5, 584.8,584.9 & Procedure code 39.95	N17.0, N17.1, N17.2, N17.8, N17.9 & Procedure codes 5A1D60Z, 5A1D70Z, 5A1D80Z, 5A1D90Z, 5A1D00Z
	Mechanical ventilation	Procedure codes 96.70, 96.71, 96.72, 96.04	5A1935Z, 5A1945Z, 5A1955Z, 0BH17EZ, 0BH18EZ
	Pressor use	00.17, 99.29	3E030XZ, 3E033XZ, 3E040XZ, 3E043XZ, 3E050XZ, 3E053XZ, 3E060XZ, 3E063XZ

[*] *Data source*: International Classification of Disease, Ninth Revision and Tenth Revision, Clinical Modification (ICD-9-CM and ICD-10-CM); Elixhauser comorbidity coding algorithm.
ICD-9 CM, the International Classification of Diseases, Ninth Revision, Clinical Modification; *ICD-10 CM*, the International Classification of Diseases, Tenth Revision, Clinical Modification; *NAFLD*, Non-Alcoholic Fatty Liver Disease; *N/A*, not available; *TIA,* transient ischemic attack.

[a] Due to the transition from the *ICD-9-CM* code to the *ICD-10-CM code* which happened on October 1st, 2015, *ICD-9-CM* diagnostic codes were used for case queries from January 1st, 2000, to September 30th, 2015.
[b] *ICD-10-CM* diagnostic codes were applied for case query from October 1st, 2015, to December 31st, 2019.

Supplementary Table S2 Elixhauser Comorbidity Index variables

Number	Variable
1	Congestive Heart Failure
2	Cardiac Arrhythmia
3	Valvular disease
4	Pulmonary circulation disorder
5	Periphery vascular disorder
6	Hypertension, uncomplicated
7	Paralysis
8	Other neurological disorders
9	Chronic pulmonary disease
10	Diabetes, uncomplicated
11	Diabetes, complicated
12	Hypothyroidism
13	Renal failure
14	Liver disease
15	Peptic ulcer disease excluding bleeding
16	AIDS/HIV
17	Lymphoma
18	Metastatic cancer
19	Solid tumor without metastasis
20	Rheumatoid arthritis/collagen vascular
21	Coagulopathy
22	Obesity
23	Wight loss
24	Fluid and electrolyte disorders
25	Blood loss anemia
26	Deficiency anemia
27	Alcohol abuse
28	Drug abuse
29	Psychoses
30	Depression
31	Hypertension, complicated

Supplementary Table S3 Case number of total sepsis discharges, concurrent NAFLD case numbers, and percentage of NAFLD among sepsis discharges from 2000 to 2019 in the US[*]

Year	Sepsis discharges (Age >=18)	Comorbid NAFLD cases among sepsis discharges	NAFLD/ sepsis (%)
2000	311,650	3,759	1.2
2001	319,734	3,922	1.2
2002	327,160	4,394	1.3
2003	369,823	5,421	1.5
2004	431,566	6,751	1.6
2005	506,459	7,321	1.4
2006	579,485	9,078	1.6
2007	644,698	10,708	1.7
2008	751,918	10,315	1.4
2009	779,997	14,641	1.9
2010	873,835	17,760	2.0
2011	1,034,863	22,715	2.2
2012	1,119,780	27,370	2.4
2013	1,281,515	32,785	2.6
2014	1,495,865	41,395	2.8
2015[a]	1,759,905	53,620	3.0
2016	1,897,269	67,320	3.5
2017	2,082,494	76,725	3.7
2018	2,215,695	86,070	3.9
2019	2,274,200	94,525	4.2
All	21,057,911	596,595	2.8

NAFLD, Non-Alcoholic Fatty Liver Disease; *US*, United States

[*]Data source: The U.S. Census Bureau; National Inpatient Sample (NIS), 2000-2019.

[a] Year 2015, from 2015 Quarter 1 to Quarter 3 (2015 Q1-Q3), there were 1,306,620 sepsis discharges, with 38,015 concurrent NAFLD cases; in NIS 2015 Quarter 4(2015 Q4), there were 453,285 sepsis discharges, with 15,605 concurrent NAFLD cases.

Supplementary Table S4 Results of the multivariate logistic regression model assessing the impact of NAFLD on sepsis inpatient all-cause mortality

Parameters	aOR	95% CI		P Value
		Lower	Upper	
NAFLD	1.19	1.07	1.32	0.001
BMI (kg/m^2)				
20-24.9	1(Reference)			
≤ 19.9	1.45	1.35	1.55	<0.001
25.0-29.9	0.84	0.75	0.93	0.001
≥ 30	0.65	0.61	0.71	<0.001
Age	1.023	1.021	1.026	< 0.001
**Sex**				
Male	1(reference)			
Female	1.02	0.97	1.07	0.41
Race				
White	1(reference)			
Black	1.06	0.98	1.14	0.125
Hispanic	0.85	0.78	0.93	0.001
Asian/Pacific Islanders/Native American/others	1.12	1.02	1.24	0.017
**Median household income**				
0-25th percentile	1 (Reference)			
26-50th percentile	0.92	0.86	0.98	0.013
51-75th percentile	0.84	0.78	0.90	<0.001
76-100th percentile	0.86	0.79	0.93	<0.001
**Insurance Status**				
Medicare	1(reference)			
Medicaid	1.15	1.05	1.26	0.002
Private	1.04	0.97	1.13	0.269
Self-pay	1.24	1.07	1.44	0.004
No charge or other	1.16	1.25	1.71	< 0.001
**Hospital Bed size**				
Small	1(reference)			
Medium	1.17	1.08	1.28	< 0.001
Large	1.35	1.25	1.45	< 0.001
**Hospital teaching status**				
Non-teaching	1(reference)			
Teaching	1.33	1.25	1.43	< 0.001
**Hospital Region**				
Northeast	1 (reference)			
Midwest	0.81	0.74	0.90	< 0.001
South	0.89	0.81	0.98	0.013
West	0.92	0.83	1.01	0.072

Other comorbidities				
Congestive Heart Failure	1.53	1.45	1.61	< 0.001
Cardiac Arrhythmias	1.52	1.44	1.59	< 0.001
Chronic Pulmonary Disease	1.02	0.97	1.07	0.482
Renal Failure	1.25	1.19	1.32	< 0.001
Paralysis	1.26	1.1	1.45	0.001
HTN	0.72	0.68	0.76	<0.001
HLD	0.71	0.67	0.74	<0.001
Metabolic syndrome	0.54	0.26	1.13	0.1
Complicated diabetes mellitus	0.90	0.85	0.96	0.001
Coagulopathy	2.41	2.28	2.55	< 0.001
Fluid and electrolytes Disorders	2.07	1.94	2.21	< 0.001
Smoking	0.81	0.76	0.85	<0.001
Alcohol abuse	1.48	1.34	1.62	< 0.001
Stroke	1.16	1.07	1.26	< 0.001

NAFLD, Non-Alcoholic Fatty Liver Disease; *aOR*, Adjusted Odds Ratio; *CI,* Confidence Interval; *BMI*, Body Mass Index; *kg/m^2*, kilogram per square meters*; HTN,* Hypertension*; HLD,* Hyperlipidemia.

Supplementary Table S5. Stratification analysis: In-hospital outcomes of patients admitted for Sepsis, severe Sepsis, Septic shock with and without steatosis

NAFLD Stage	Outcomes	Sepsis		Severe Sepsis		Septic shock	
		Adjusted OR (95% CI) *	P value	Adjusted OR (95% CI)	P Value	Adjusted OR (95% CI)	P Value
Steatosis	Died	0.65(0.53-0.81)	<0.001	0.55(0.31-0.98)	0.043	0.70(0.55-0.88)	0.002
	Type of organ dysfunction						
	Hepatic	1.12(0.89-1.41)	0.321	1.42(0.84-2.40)	0.185	1.10(0.83-1.16)	0.516
	Respiratory	0.78(0.71-0.85)	<0.001	0.77(0.64-0.92)	0.004	0.67(0.56-0.81)	<0.001
	Cardiovascular	0.85(0.77-0.94)	0.002	0.77(0.56-1.06)	0.105	1	–
	Renal	1.01(0.93-1.10)	0.805	0.96(0.81-1.13)	0.615	1.001(0.83-1.21)	0.984
	Neurological	0.70(0.61-0.79)	<0.001)	0.61(0.46-0.79)	<0.001)	0.73(0.60-0.89)	0.002
	Hematologic	1.41(0.93-2.15)	0.102	0.996(0.46-2.14)	0.93	1.58(0.77-3.25)	0.209
	Metabolic	1.17(1.06-1.29)	0.002	1.07(0.89-1.29)	0.505	1.18(0.97-1.44)	0.088
	Septic shock	0.88(0.79-0.95)	0.025	1.39(0.62-3.14)	0.42	-	-
	Procedures						
	Mechanical Ventilation	0.74(0.65-0.85)	<0.001	0.59(0.42-0.83)	0.002	0.74(0.62-0.89)	0.001
	Acute kidney injury requiring hemodialysis	0.95(0.76-1.23)	0.71	0.63(0.31-1.30)	0.211	1.20(0.92-1.57)	0.177
	Pressor use	0.89(0.71-1.12)	0.311	0.84(0.38-1.88)	0.677	0.93(0.73-1.18)	0.54

NAFLD, Non-Alcoholic Fatty Liver Disease; *OR*, Odds Ratio; CI, Confidence Interval.

* The results were adjusted for age, sex, race, median house income percentile, insurance status, hospital region, hospital bed size, hospital teaching status, BMI, congestive heart failure, cardiac arrhythmia, chronic pulmonary disease, diabetes(complicated), renal failure, hypertension, hyperlipidemia, metabolic syndrome, paralysis, coagulopathy, fluid and electrolytes disorder, smoking, alcohol use, and stroke.

Supplementary Table S6. Discharge disposition of patients admitted for sepsis with and without NAFLD groups

**Discharge disposition**	**Overall**	**Sepsis-non-NAFLD**	**Sepsis-NAFLD**	**RRR (95% CI)**	***P-value****
Home with self-care or home health care	64.8%	64.6%	69.4%	Baseline outcome	
Transfer to a short-term hospital	3.5%	3.5%	4.4%	1.24 (1.08-1.42)	0.002
Transfer other[a]	29.6%	29.8%	24.4%	0.86 (0.80-0.93)	< 0.001
Against Medical Advice	2.1%	2.1%	1.8%	0.82 (0.63-1.06)	0.129
Others[b]	0.02%	0.02%	0.02%	4.85 (1.25-18.78)	0.022

NAFLD, Non-Alcoholic Fatty Liver Disease; *SNF*, skilled nursing facility; *%*, percentage; *RRR*, Relative Risk Ratio; *CI*, Confidence Interval.

*= Adjusted P-value. The results were adjusted for age, sex, race, insurance status, hospital region, hospital bed size, hospital teaching status, median household income percentile, BMI, congestive heart failure, cardiac arrhythmia, chronic pulmonary disease, hypertension, hyperlipidemia, diabetes(complicated), metabolic syndrome, renal failure, paralysis, coagulopathy, fluid and electrolytes disorder, smoking, alcohol use, and stroke.

[a] = Including Skilled Nursing Facility (SNF), Intermediate Care Facility (ICF), Another Type of Facility.

[b] = Including discharge alive, destination unknown, and missing.

Supplementary Table S7. Mediation analysis of the role of metabolic syndrome on the impact of NAFLD on sepsis inpatient mortality.

	Sepsis inpatient mortality						
	Total effect		Direct effect		Indirect effect		Mediation or suppression, %
**Mediator**	β (95% CI)	*P* value*	β (95% CI)	*P* value	β (95% CI)	*P* value	
**Metabolic syndrome**							
NAFLD	0.0143848 (0.0098-0.01887)	<0.001	0.0144303 (0.0099427-0.0188179)	<0.001	-0.0000455 (-0.0000896-0.0000134	0.043	0.3

NAFLD, Non-Alcoholic Fatty Liver Disease; *CI*, Confidence Interval.

*= Adjusted P-value. The results were adjusted for age, sex, race, insurance status, hospital region, hospital bed size, hospital teaching status, obesity, congestive heart failure, cardiac arrhythmia, chronic pulmonary disease, hypertension, hyperlipidemia, diabetes(complicated), renal failure, paralysis, coagulopathy, fluid and electrolytes disorder, alcohol use, and stroke.

Supplementary Table S8 Sensitivity analysis: Results of the multivariable logistic regression model assessing the impact of NAFLD on sepsis inpatient all-cause mortality after excluding severe inflammatory comorbidities, advanced cardiovascular disease, and chronic kidney failure

Parameters	aOR	95% CI		P Value
		Lower	Upper	
NAFLD	1.28	1.04	1.58	0.018
BMI (kg/m^2)				
20-24.9	1(Reference)			
≤ 19.9	1.66	1.44	1.92	<0.001
25.0-29.9	0.73	0.58	0.93	0.009
≥ 30	0.72	0.61	0.84	<0.001
Age	1.04	1.03	1.04	< 0.001
Sex				
Male	1(reference)			
Female	0.93	0.84	1.03	0.167
Race				
White	1(reference)			
Black	0.91	0.78	1.05	0.203
Hispanic	0.79	0.67	0.94	0.008
Asian/Pacific Islanders/Native American/others	1.11	0.92	1.35	0.272
Median household income				
0-25th percentile	1 (Reference)			
26-50th percentile	0.95	0.83	1.09	0.013
51-75th percentile	0.79	0.67	0.94	0.008
76-100th percentile	0.80	0.69	0.94	0.006
Insurance Status				
Medicare	1(reference)			
Medicaid	1.35	1.13	1.61	0.001
Private	0.92	0.79	1.08	0.325
Self-pay	1.56	1.22	1.99	<0.001
No charge or other	1.49	1.09	2.05	0.013
Hospital Bed size				
Small	1(reference)			
Medium	1.08	0.93	1.25	0.310
Large	1.32	1.16	1.51	< 0.001
Hospital teaching status				
Non-teaching	1(reference)			
Teaching	1.52	1.34	1.72	< 0.001
Hospital Region				
Northeast	1 (reference)			
Midwest	0.86	0.72	1.02	0.078
South	0.97	0.83	1.13	0.704
West	0.93	0.79	1.09	0.394
Other comorbidities				
Cardiac Arrhythmias	1.66	1.49	1.86	< 0.001

Paralysis	1.26	0.96	1.64	0.091
HTN	0.70	0.62	0.78	<0.001
HLD	0.75	0.66	0.85	<0.001
Metabolic syndrome	1.04	0.34	3.12	0.07
Complicated diabetes mellitus	0.76	0.65	0.88	<0.001
Coagulopathy	2.95	2.62	3.32	<0.001
Fluid and electrolytes Disorders	2.26	1.98	2.58	<0.001
Smoking	0.74	0.66	0.85	<0.001
Alcohol abuse	1.86	1.58	2.20	<0.001

NAFLD, Non-Alcoholic Fatty Liver Disease; *aOR*, Adjusted Odds Ratio; *CI,* Confidence Interval; *BMI*, Body Mass Index; *kg/m²*, kilogram per square meters*; HTN,* Hypertension*; HLD,* Hyperlipidemia.

Supplementary Figure S1

Figure caption for Supplementary Figure. 1.

From the year 2000 to the year 2019, the overall length of stay for sepsis patients who were discharged alive declined from 9.4 days in 2000 to 6.8 days in 2019; Similar trends were observed for both the NAFLD and non-NAFLD group patients. There was no significant difference regarding the length of stay between these two groups in each study year.

Supplementary Figure 1. Trends of Adjusted In-Hospital Length of Stay of Sepsis Patients who were discharged alive, With and Without NAFLD from 2000 to 2019 in the US

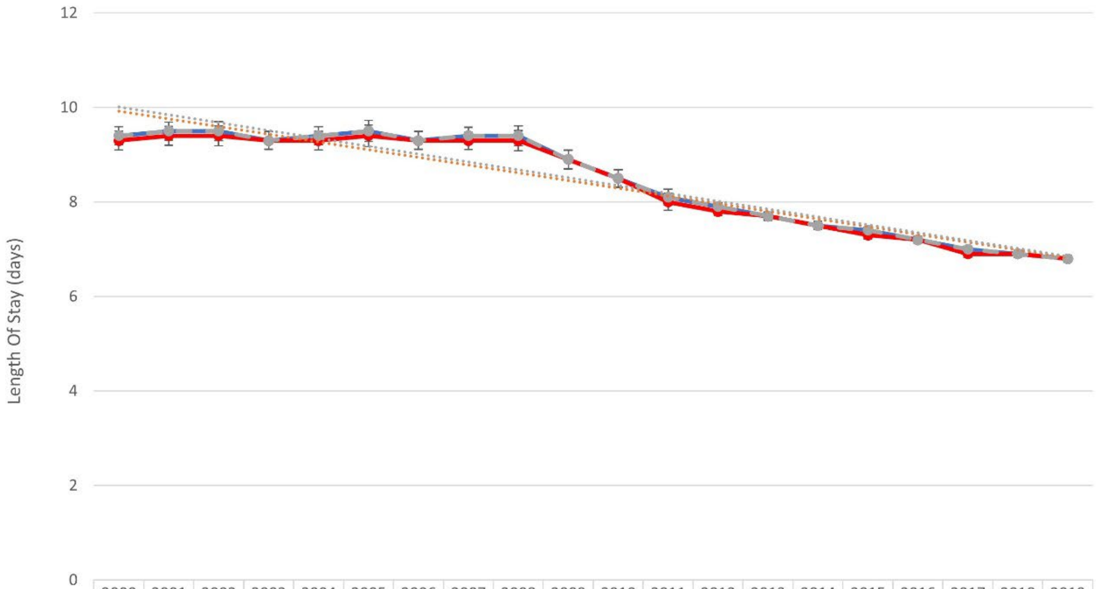

	2000	2001	2002	2003	2004	2005	2006	2007	2008	2009	2010	2011	2012	2013	2014	2015	2016	2017	2018	2019
Sepsis-All	9.4	9.5	9.5	9.3	9.4	9.5	9.3	9.4	9.4	8.9	8.5	8.1	7.9	7.7	7.5	7.4	7.2	7.0	6.9	6.8
Sepsis-NAFLD	9.3	9.4	9.4	9.3	9.3	9.4	9.3	9.3	9.3	8.9	8.5	8.0	7.8	7.7	7.5	7.3	7.2	6.9	6.9	6.8
Sepsis-non-NAFLD	9.4	9.5	9.5	9.3	9.4	9.5	9.3	9.4	9.4	8.9	8.5	8.1	7.9	7.7	7.5	7.4	7.2	7.0	6.9	6.8

Calendar Year

Sepsis-All Sepsis-NAFLD Sepsis-non-NAFLD Linear (Sepsis-NAFLD) Linear (Sepsis-non-NAFLD)

Supplementary Figure S2

Figure caption for Supplementary Figure. 2.

Mediation analysis of metabolic syndrome in the association of NAFLD and sepsis in-hospital all-cause mortality. The mediation structural model was adjusted for age, sex, race, insurance status, hospital region, hospital bed size, hospital teaching status, obesity, congestive heart failure, cardiac arrhythmia, chronic pulmonary disease, hypertension, hyperlipidemia, diabetes(complicated), renal failure, paralysis, coagulopathy, fluid and electrolytes disorder, alcohol use, and stroke. β is the standardized regression coefficient. A *P* value less than 0.05 was considered to be statistically significant.

Supplementary Figure.2 Mediation analysis of metabolic syndrome on the impact of NAFLD on sepsis in-hospital mortality.

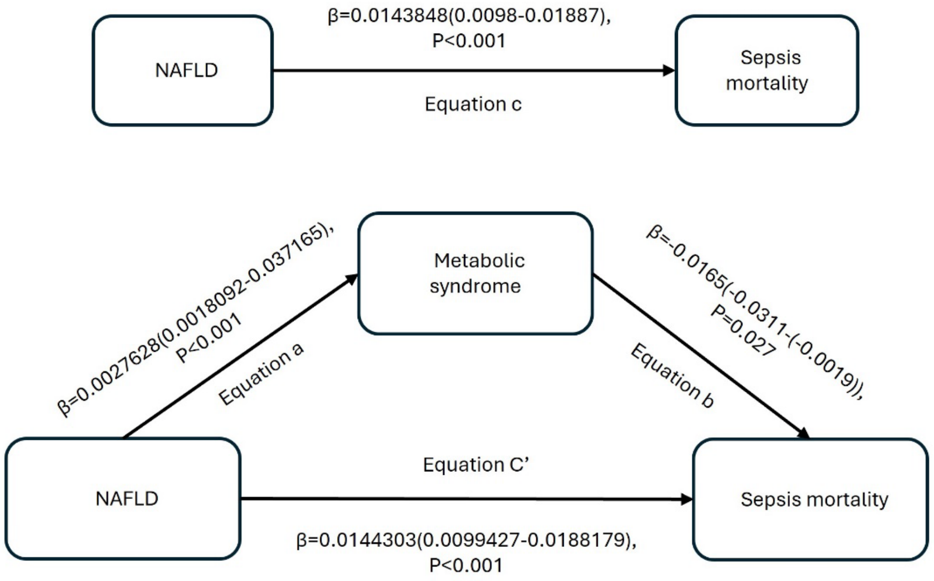

Mediation effect: β=-0.0000455 (-0.0000896-0.0000134); P=0.043, Proportion of mediation=0.3%.
